# Genetic monitoring of a long-term, large-scale experimental steelhead supplementation program

**DOI:** 10.1371/journal.pone.0339458

**Published:** 2025-12-22

**Authors:** Donald M. Van Doornik, Barry A. Berejikian, Andrew M. Claiborne

**Affiliations:** 1 Conservation Biology Division, Northwest Fisheries Science Center, National Marine Fisheries Service, National Oceanic and Atmospheric Administration, Port Orchard, Washington, United States of America; 2 Environmental and Fisheries Sciences Division, Northwest Fisheries Science Center, National Marine Fisheries Service, National Oceanic and Atmospheric Administration, Port Orchard, Washington, United States of America; 3 Washington Department of Fish & Wildlife, Olympia, Washington, United States of America; University of Nevada, Reno, UNITED STATES OF AMERICA

## Abstract

Declining salmonid populations often prompt the use of captive-reared fish to supplement wild stocks, but such programs risk negative genetic and ecological impacts. We evaluated six steelhead (*Oncorhynchus mykiss*) populations in the Hood Canal watershed, Puget Sound, Washington, including three supplemented and three unsupplemented control populations, over the span of 17 years to assess the effects of supplementation on several population genetics metrics. This program uniquely allowed natural spawning to occur before removing eyed eggs from redds for captive rearing, and later release as smolts or adults. Key genetic metrics—expected heterozygosity, allelic richness, and effective population size—remained stable from before to after supplementation in both the supplemented and non-supplemented populations. Parentage analyses confirmed successful reproduction by captively reared adults after they were released into the wild. These findings suggest that natural spawning prior to captive rearing, among other aspects of the program, lessened the genetic risks typically associated with artificial propagation such as loss of genetic diversity, or a reduction in effective population size. Our results highlight the potential for carefully designed supplementation programs to conserve genetic diversity and maintain effective population sizes in threatened steelhead populations.

## Introduction

Supplementation programs, where captive-reared fish are introduced into wild populations to enhance natural production and increase abundance, have been widely implemented as a conservation strategy for declining salmonid populations. However, previous studies have highlighted several potential drawbacks of supplementation efforts, including genetic and ecological risks that may outweigh the intended benefits [1–7]. Understanding these risks is critical because the long-term success of supplementation programs depends not only on their ability to increase population sizes but also on their capacity to maintain the genetic health of these populations. Careful genetic monitoring is therefore essential to assess the effectiveness of these efforts and prevent unintended harm.

One concern in supplementation is the potential reduction in effective population size (*N*_*e*_). Unlike census size, *N*_*e*_ reflects the genetic processes of a population and is defined as the size of an idealized population that would undergo genetic change (measured as either inbreeding or random genetic drift) at the same rate as the actual population [8]. It is typically smaller than the census population size, due to variance in reproductive success and sex ratios. A reduction of effective population size can potentially lead to loss of long-term fitness through generations due to loss of genetic diversity or increased inbreeding [9], as demonstrated in recent studies [7,10].

Previous studies of supplementation programs have provided important insights into the genetic effects of hatchery interventions, particularly in documenting differences in reproductive success between captive and naturally reared fish. However, a common limitation of these studies is the lack of unsupplemented control populations. By focusing solely on supplemented populations, it can be difficult to determine whether any observed genetic changes are caused by supplementation, or if they can be attributed to other natural freshwater or marine conditions influencing survival patterns and population abundance. In addition, many past studies of supplementation focused on the relative reproductive success between captive and naturally reared fish, but did not evaluate before and after effects of supplementation on the population as a whole. Before-After Control-Impact (BACI) studies are a robust methodological approach for assessing the effects of changes by comparing data collected before and after an event, both at impacted sites and control sites [11,12]. We sought to incorporate a BACI design in a study of the effects of a supplementation program on several natural populations.

Our research focused on six steelhead (*Oncorhynchus mykiss*) populations within the Hood Canal Watershed in Puget Sound, Washington, with three populations receiving supplementation and three serving as unsupplemented controls. The hatchery programs developed for this study were designed to be small to minimize density-dependent ecological effects on the natural populations [13]. However, creating small captive populations can result in the Ryman-Laikre effect [9], where a very small number of parents become over-represented in the population, and may ultimately result in reduced genetic diversity [14,15]. One potential way to avoid this problem is to create a captive population by sampling a small number of individuals from each of a large number of families. In the present study, a portion of the eyed embryos in naturally constructed steelhead redds were sub-sampled, so that a greater number of families would be represented in the captive populations compared with the alternative approach of artificially spawning a few adults to produce the same number of offspring. Subsampling naturally constructed redds also allowed natural selection on spawning location, timing and redd characteristics, and sexual selection (i.e., mate choice) to occur without human intervention. Collected embryos were incubated and raised in captivity until they were ready for release back into the rivers as either smolts or sexually mature adults.

The objectives of our study were to 1) determine whether conservation hatchery programs caused changes in measures of genetic diversity over approximately four generations in supplemented steelhead populations by comparing them to unsupplemented, control populations from the same region, and 2) document the reproductive success in nature of released, captively-reared adult steelhead. Through this research, we aim to contribute to the growing body of literature on salmonid supplementation and provide insights to help guide the future management and conservation of steelhead populations.

## Materials and methods

### Study populations

**Fig 1:**
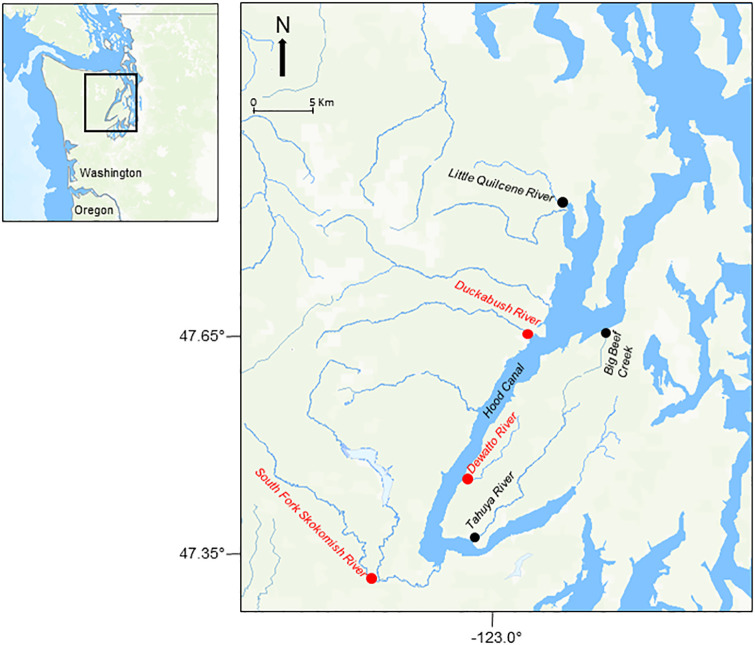
Locations of the six Hood Canal rivers monitored in this study. The Dewatto, Duckabush, and South Fork Skokomish rivers were supplemented (shown in red), whereas the Little Quilcene and Tahuya rivers, and Big Beef Creek were monitored as controls. Map source: Commission for Environmental Cooperation (CEC). North American Atlas – North American Lakes and Rivers, 2023 [shapefile]. Available at: https://www.cec.org/north-american-environmental-atlas/lakes-and-rivers-2023/.

Our study populations came from six rivers within the Hood Canal Watershed (Fig 1). Three of the populations were monitored as controls (Big Beef Creek, Little Quilcene and Tahuya rivers) and three of the populations were supplemented with captive-reared fish (Dewatto, Duckabush, and South Fork Skokomish rivers). Details of the supplementation program can be found in Berejikian et al. [13]. Here we provide a brief description of the program as it relates to our study. The captive group used for supplementation was derived from eight years of annual collections of eyed eggs extracted from steelhead redds (nests) using hydraulic methods [44]. Eggs were collected from each redd until either the desired quantity was obtained or the entire redd had been exhaustively sampled. The desired number of eggs was dynamically adjusted based on the total egg collection goal for each population, the number of eggs previously collected, and the number of redds expected during the remainder of the spawning season. The targeted number of eggs per redd was typically between 200−400. These fish were raised in captivity to be released back into their natal river, primarily as either age-2 smolts from every brood year or as age-4 or age-5 adults from every other year (except for brood year 2008 in the Duckabush River). A small number of age-1 smolts and age-6 adults were also released. In this way, all spawning occurred naturally within the rivers. We avoided sampling any redds that were observed to have been made by captively-reared fish. Thus, the proportion of redds sampled was much higher during the first four years of the study, before any of the captively-reared fish were spawning naturally in the rivers, especially for the Dewatto and Duckabush rivers (Table 1).

**Table 1:** Number of redds (by brood year) used for egg collection to produce captive-reared steelhead and the corresponding number of fish released into each supplemented population as smolts or mature adults. Most releases were expected to contribute as four-year-olds, with some fish contributing as five- or six-year-olds. Data are from Berejikian et al. [13].

Population/ Brood year	Redds observed	Redds sampled	Smolts	Adults
Dewatto				
2007	17	16	7,375	252
2008	30	18	6,807	0
2009	9	9	6,571	259
2010	8	7	4,956	0
2011	57	19	5,272	261
2012	34	12	6,183	0
2013	130	16	6,473	260
2014	17	12	4,239	0
Duckabush				
2007	10	6	1,574	199
2008	11	8	4,671	135
2009	8	1	0	0
2010	18	6	1,883	196
2011	77	12	2,550	0
2012	64	10	4,782	221
2013	34	8	4,713	0
2014	39	8	1,700	0
South Fork Skokomish				
2007	235	35	27,838	71
2008	155	50	20,729	0
2009	280	40	26,642	257
2010	169	38	23,989	0
2011	243	36	22,717	357
2012	307	30	27,258	0
2013	668	39	18,005	188
2014	390	42	14,769	0

### Sample collection for genetic monitoring

We collected samples for genetic monitoring using various methods. From 2006 to 2023, naturally-produced (hereafter, ‘natural’), juvenile *O. mykiss* were collected annually via screw traps placed in the lower reaches of the rivers from April through June. In addition, samples were also collected every year (excluding 2023) using hook-and-line sampling from late August through early September across the section of the rivers accessible to steelhead spawners, targeting juvenile *O. mykiss*, which represent a combination of progeny from resident and steelhead mothers [16]. For each fish sampled, basic metrics were recorded such as length, weight, and smoltification status, and a small fin clip was taken and preserved in 95% ethanol. In addition, several scales were removed from each juvenile fish and mounted on gummed scale cards that were sent to the Washington Department of Fish and Wildlife Fish Aging Laboratory to determine the age of the fish sampled. In an attempt to standardize parr collections to represent juveniles before they outmigrate to sea, we did not include hook-and-line caught fish that were greater than 170 mm in length. A fin clip was also taken from all captive-reared fish released as adults (hereafter referred to as the Adult Release Group (ARG)) and a subsample of the fish released as smolts (hereafter referred to as the Smolt Release Group (SRG)).

### Aging

Scale analysis was used to determine age and birth year (i.e., brood year) of juveniles collected via the screw traps and hook-and-line sampling. Scale aging was unnecessary for fish in the SRG or ARG groups, as their birth years were known. Acetate impressions were made of each card using a heated hydraulic press and viewed using a Realist^©^ microfiche or Leica S9i^©^ digital microscope camera. We identified alternating zones of tightly and widely spaced circuli, termed annuli, to describe the number of winters or years a fish has lived, similar to [17].

### Genotyping

Genomic DNA was extracted from the fin clip samples using Promega Wizard DNA Purification Kits (Promega Corporation). Polymerase chain reactions (PCR) were performed to amplify 15 microsatellite loci: Ocl1 [18], Oke4 [19], Oki23 [20], Ogo4 [21], Omy1001, Omy1011 [22], Omy77 [23], Omy7 [24], Oneu14 [25], Ots100 [26], Ots3, Ots4 [27], Ssa289 [28], Ssa407, and Ssa408 [29]. The PCR products were then analyzed via capillary electrophoresis and sized using an Applied Biosystems 3100 or 3730 genetic analyzer (Life Technologies, Grand Island, NY). Allele calls between the two platforms were standardized by genotyping control samples on both systems. Individual genotypes were determined using GeneScan and Genotyper software or Genemapper software (Life Technologies).

### Data analyses

All of the rivers in our study contain cutthroat trout (*O. clarkii*), which readily hybridize with *O. mykiss*. Despite visually inspecting fish for hybrid characteristics at the time of sampling and excluding any fish displaying such traits, hybrids were still found in our samples, as phenotypic characters are not reliable for identifying all hybrids. Suspected hybrids, belonging to any filial generation, were identified by the presence of cutthroat trout specific alleles in three of our loci (*Oke4*, *Ot3*, *Ots100*). Individuals expressing any of these alleles were genotyped for four additional loci (*OCC-34*, *OCC-35*, *OCC-42*, *OM-47*) that were specifically developed to distinguish among *O. mykiss*, cutthroat trout, and their hybrids [30]. The presence of cutthroat troutspecific alleles at any of these loci was considered evidence of cutthroat trout ancestry, and such individuals were excluded from further analyses.

Sampling juveniles from within a river carries the risk of collecting individuals that are highly related to each other as full siblings, which could bias allele frequency estimates [31]. Although many researchers exclude potential siblings prior to downstream analyses, Waples and Anderson [32] caution that this practice can introduce new problems, including reduced statistical power and precision due to smaller sample sizes, as well as inflated estimates of effective population size. Therefore, we did not remove any putative siblings from our samples.

We measured several statistics frequently used to quantify the magnitude of genetic diversity within populations. Genetic variability within a sample was assessed using expected heterozygosity, calculated in GenAlEx [33]. Allelic richness, which accounts for sample size, was determined using HP-RARE [34], using a minimum sample size of eight genes. The resulting metric values (and standard errors) for the natural samples were averaged across brood years within each population and grouped by supplementation phase (pre, during, and post). Metrics were calculated for each brood year sample to assess potential changes in supplemented populations resulting from the release of captive-reared fish. These same metrics were also calculated for control populations to evaluate whether any observed changes could be attributed to factors unrelated to the supplementation program. Samples from captive-reared fish released as adults or smolts were analyzed separately from natural juvenile samples of the same brood year collected in rivers.

We estimated the effective number of breeders (*N*_*b*_*)* for each of our brood year samples using the program NeEstimator [35] with the linkage disequilibrium model and jackknife confidence intervals. We excluded singleton alleles, which is the most effective way to minimize upward bias from rare alleles [36]. The *N*_*b*_ values for natural samples were averaged across brood years within each population and supplementation phase using harmonic means. For species such as steelhead with overlapping generations in their life history, the effective population size estimate represents the effective number of breeders per year (*N*_*b*_) rather than per generation [37]. The relationship between *N*_*b*_ and *N*_*e*_ can be expressed by the equation, *N*_*e*_ = g *N*_*b*_, where g is the generation length, or average age at spawning [38]. We calculated *N*_*e*_ for each brood year using this formula with g = 4.1, as determined from spawning data for five Puget Sound steelhead populations [39].

### Supplementation effects

All metrics were tested for significant changes between the before and after supplementation periods of the study. Brood years from 2004–2010 represented the “before” phase, when only naturally produced steelhead were spawning in the rivers. Brood years from 2011–2018 represented the “during” phase when captive-reared fish were spawning in the rivers, and brood years 2019–2021 represented the “after” phase, when all spawning occurred by naturally-reared steelhead once again. The before and after values for each river for expected heterozygosity, allelic richness, and the effective number of breeders were tested for significant differences using Mann-Whitney U tests. Tests were considered significant if *P* < 0.05.

All of the ARG fish released into the supplemented streams were sampled, which provided the opportunity to conduct a parentage analyses to estimate the contribution of ARG adults to the F1 juvenile populations. Using the program FRANz [40], we attempted to match the adults to their potential offspring in our juvenile samples. Parent–offspring relationships were primarily identified using exclusion-based assignments, in which no loci mismatches occurred among parent–offspring pairs or trios. When exclusion-based matches were not possible, likelihood-based assignments were accepted if there were no more than two mismatching loci. We then calculated the proportion of our total sample of juveniles that were produced by ARG fish for the relevant brood years. For this calculation, each juvenile was counted twice, once for its father and once for its mother, to account for situations where only one of its parents was an ARG fish.

## Results

### Sample sizes

After excluding all *O. mykiss* – *O. clarkii* hybrids, the sample sizes for each brood year collection ranged from 6 to 389 (S1 Table). Hybrids were most prevalent in the Little Quilcene and Big Beef rivers, accounting for 51.9% and 41.1% of the samples, respectively. The Dewatto River followed with 11.6%, while the Tahuya (5.3%), Duckabush (1.1%), and South Fork Skokomish (0.4%) rivers showed lower hybrid proportions. It is important to note that, as aforementioned, all fish were visually inspected for hybrid traits at the time of capture, and those suspected of being hybrids were excluded from sampling. Consequently, the hybrid percentages identified through genetic analyses likely underestimate the true prevalence of hybrids in these populations.

### Aging

Natural fish within each population were grouped together by brood year, as determined by scale aging. Ages ranged from 0–6 years. Over 97% of hook-and-line caught fish were one (70.7%) or two (26.7%) years old, and about 90% of those captured in the screw traps were one (32.5%), two (57.0%), or three (10.0%) years old.

### Genetic metrics

#### Natural populations.

**Fig 2:**
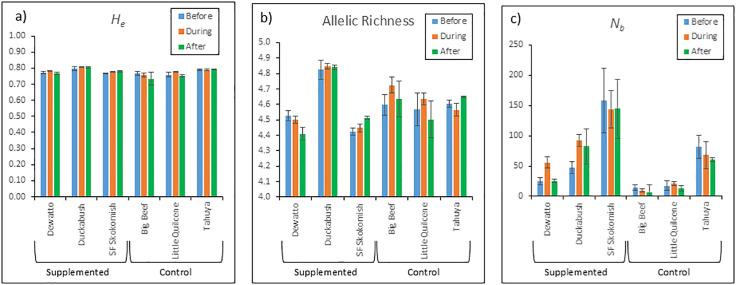
Mean values and standard errors of genetic metrics calculated before, during-, and after supplementation in three supplemented populations (Dewatto, Duckabush and South Fork Skokomish rivers) and three unsupplemented control populations (Big Beef Creek, Little Quilcene and Tahuya rivers). Metrics include a) expected heterozygosity, b) allelic richness, and c) effective number of breeders per year.

The Duckabush and South Fork Skokomish river natural populations had small increases in both expected heterozygosity and allelic richness values from pre- to post-supplementation, whereas the Dewatto River population decreased, but none of the changes were statistically significant (*P* > 0.05; Fig 2). Likewise, the control populations showed both increases and decreases in these metrics, none of which differed significantly over the same period.

For the effective number of breeders per year (*N*_*b*_), the Duckabush population showed an increase, the Dewatto population remained essentially unchanged, and the South Fork Skokomish population exhibited a slight decrease; however, none of these changes were statistically significant (P < 0.05; Fig 2). During the same period, *N*_*b*_ estimates for the control streams all declined, but these changes were also not significant. The *N*_*b*_ for the Dewatto and Duckabush populations increased during supplementation but declined post-supplementation. While the Duckabush population’s pre-supplementation harmonic mean appeared substantially lower than its post-supplementation value (Fig 2), this was skewed by a single low estimate for brood year (BY) 2008 (*N*_*b*_ = 17.8, CI = 2–∞), likely due to a small sample size (N = 6). Other *N*_*b*_ estimates from the pre-supplementation period were much higher (*N*_*b*_ = 94.8–193.0) and more consistent with post-supplementation values. For the South Fork Skokomish BY 2008 sample, the *N*_*b*_ estimate was negative, suggesting that the observed variance was likely due entirely to sampling variance [41]. This estimate was excluded from the harmonic mean calculation.

Post-supplementation *N*_*e*_ values ranged from 10.3 to 1077.5 (S1 Table). Based on harmonic mean calculations, the South Fork Skokomish population had the highest post-supplementation *N*_*e*_ among the supplemented populations (592.8), followed by Duckabush (337.7) and Dewatto (105.0). Among the control populations, Tahuya had the highest *N*_*e*_ (248.7), followed by Little Quilcene (52.9) and Big Beef (24.7).

#### Natural versus ARG and SRG.

Comparisons within brood years, across populations for which we had ARG or SRG samples, showed little difference in expected heterozygosity, and allelic richness values (S1 Table). However, *N*_*b*_ values were consistently higher in the natural samples, which is expected since the captive groups represent only a subset of the total population. For example, in the Duckabush population for brood year 2007, *N*_*b*_ was estimated at 47.5 for the natural population, but only 3.3 and 3.0 for the ARG and SRG groups, respectively.

#### Adult release group reproductive success.

The proportional contribution of ARG fish varied among populations and brood years (Table 2). Not surprisingly, years where the greatest number of adult fish were released were years with the greatest proportion of juveniles produced by ARG parents. ARG fish contributed the most to the Dewatto River population, averaging 31.2% across all brood years, followed by contributions of 18.5% to Duckabush and 13.7% to the South Fork Skokomish populations. It is important to note that in rivers with high proportions of cutthroat trout and hybridization, we likely underestimated the reproductive success of some captively-reared adults that may have spawned with cutthroat trout.

**Table 2:** Percent of juvenile samples produced by captive-reared steelhead released as mature adults, in each brood year for each population.

Brood year	Dewatto	Duckabush	South Fork Skokomish
2011	54.4%	31.8%	8.8%
2012	6.1%	40.7%	2.0%
2013	54.5%	1.6%	22.2%
2014	20.0%	24.1%	na
2015	36.7%	na	33.3%
2016	8.9%	25.7%	2.3%
2017	53.6%	4.3%	13.8%
2018	15.3%	0.9%	na

## Discussion

This study demonstrates that the steelhead supplementation program, through its emphasis on natural spawning, did not cause a reduction in the genetic diversity or effective population size of supplemented populations. Parentage analyses confirmed that captive-reared fish successfully reproduced in the supplemented rivers, contributing over half of the juvenile production in some years. Expected heterozygosity, allelic richness, and *N*_*b*_ showed no significant declines from before to after supplementation. Instead, we observed stability in these metrics, indicating that the supplementation program did not compromise the populations’ genetic health. Although our post-supplementation monitoring covered only three years, any harmful effects of the program would be expected to appear quickly, had they occurred. Likewise, the control populations did not experience any significant changes in these metrics.

Our findings align with previous studies emphasizing the benefits of collecting juveniles or eggs instead of adults as a broodstock source, thus avoiding any breeding within a hatchery. For example, a study on a different Hood Canal River, which also utilized natural spawning to create supplementation broodstock, did not observe deleterious genetic effects in the total population [42]. In contrast, studies of steelhead programs that relied on artificial spawning generally found different outcomes. For example, Christie et al. [4] found lower levels of genetic diversity and effective population size in hatchery-reared steelhead that were used in a supplementation program. When released into the river targeted for supplementation, those fish reduced the effective population size of the total population by nearly two-thirds. Similarly, Smith et al. [7] found that hatchery-origin fish introduced to supplement a population exhibited lower genetic diversity and effective population size than natural-origin fish. Although they did not perform a before-and-after analysis to assess overall population effects, their evidence of successful reproduction among hatchery-origin fish suggests a likely negative impact on the population’s genetic integrity.

The supplementation strategy ensured that all reproduction occurred via spawning in the study rivers and streams, which likely contributed to maintaining genetic diversity in the supplemented populations. Embryo collections removed a portion of the naturally spawned offspring for captive rearing and allowed siblings of the captively reared steelhead to remain in their natal rivers. The embryos in a single steelhead redd are frequently the offspring of more than just a single pair of parents [43], which means that a greater number of families were represented in the captive populations than would have been possible had a few adults been collected and spawned [44]. Furthermore, natural spawning allowed for more diverse mating combinations compared to artificial spawning [45], including the potential for resident males to spawn with steelhead females [46], which is precluded by artificially spawning anadromous pairs. While we can not attribute the maintenance of genetic diversity in the supplemented populations to any particular factor, the emphasis on reproduction occurring in the natural streams is consistent with maintenance of genetic diversity metrics comparable to the non-supplemented control populations.

Previous analyses of supplementation programs have found that compared with natural-origin steelhead, hatchery-reared steelhead typically have lower relative reproductive success, decreases in fitness, reductions of genetic diversity, and an increase in inbreeding [1–3,5,47]. While we could not specifically assess reproductive success or fitness in our study populations, our parentage analyses confirmed that the captively reared adults did successfully produce offspring in substantial proportions. It is important to note that those results only account for the captively reared fish released as adults. Fish released as smolts were also likely contributors to the populations, though the extent of their contribution is uncertain. Regardless, our estimates of genetic diversity showed no detectable changes caused by the supplementation program.

Berejikian et al. [13] documented a temporary increase in census population sizes as a result of the study’s supplementation program, followed by declines after supplementation ended, leaving population sizes slightly above pre-supplementation levels. Our *N*_*b*_ estimates for the Dewatto and Duckabush populations followed a similar pattern (Fig 2), whereas the South Fork Skokomish River population showed a slight decline. Notably, the South Fork Skokomish River population also exhibited the smallest change in census population size in the study by Berejikian et al. [13], and our findings suggest that this change was insufficient to increase the population’s effective size. However, none of the observed changes in *N*_*b*_ from pre- to post-supplementation for these populations were statistically significant, indicating that the supplementation program had minimal impact on effective size in all three populations.

Our study gave us the opportunity to evaluate the *N*_*e*_ values of these populations and compare them to expected thresholds for healthy populations. Maintaining an *N*_*e*_ above 50 is commonly regarded as necessary for short-term population viability by avoiding the effects of inbreeding depression [48,49]. All of the post-supplementation mean *N*_*e*_ estimates from the natural samples were above the threshold value of 50 with the exception of the Big Beef population (mean *N*_*e*_ = 24.7). This indicates that the genetic diversity within five of these populations is likely sufficient for their short-term sustainability. However, maintaining long-term viability and evolutionary potential by minimizing genetic drift, is believed to require *N*_*e*_ values greater than 500, although some authors argue that those values should be doubled to 100 and 1000 [50]. While not all of the values we estimated were above this threshold, it is important to note our study populations are part of two larger Hood Canal steelhead genetic population groups [51]. These larger, meta-population groupings are the appropriate level of population structure to be considered when discussing long-term viability and evolutionary potential.

A key point to consider is that all of the river systems in our study include interbreeding, sympatric resident and anadromous forms of *O.mykiss*. Only embryos from anadromous (steelhead) females were collected for the captive populations. Across all of the study populations, steelhead females produce female offspring with a very low propensity to mature in freshwater (i.e., resident form), whereas male offspring have a much higher rate of residency (approximately 20−40%) under natural conditions [52] (Bush et al. 2014), possibly having been sired by resident fathers (43). The consequences of collecting and rearing would-be resident males in captivity and releasing them at age-4 or age-5 is unknown, except to note that after release they would have the opportunity to contribute to natural reproduction and likely at a larger size than if they had matured in nature. Unlike conventional steelhead hatchery programs where both parents are always anadromous, resident males likely contributed significantly to the captive populations in the present study [43]. Perhaps the contrast between typical hatchery programs and those that rely on natural production partly explains the maintenance of genetic diversity seen here. Furthermore, Van Doornik et al. [53] observed that resident *O. mykiss*, particularly males, produce anadromous offspring in both the Duckabush and South Fork Skokomish rivers. Since our Ne estimates reflect all *O. mykiss* successfully spawning within each river system, a substantial population of resident fish could significantly elevate estimates in those systems. Resident *O. mykiss* that contribute to anadromous offspring may play an essential role in stabilizing Ne within a population [54], potentially helping to offset low marine survival experienced by the anadromous populations in recent years [55].

In summary, our study provides important insights into the effects of a multi-population steelhead supplementation program on genetic diversity. The findings support the idea that careful design of supplementation programs, particularly those leveraging natural spawning, have the potential to maintain genetic diversity and lessen the genetic risks that have been associated with more conventional artificial propagation of anadromous salmonids. While the longer-term genetic effects were not assessed in this study, other programs have realized reduced genetic diversity during similar timeframes. Important, broader, conservation efforts are underway for steelhead in Hood Canal the Pacific Northwest. In cases where coincident conservation hatchery programs may be considered, genetically robust approaches to sourcing fish for captivity, developing thoughtful release strategies, and careful monitoring of supplemented and non-supplemented populations may be critical for conserving vulnerable steelhead populations and similar species.

## Supporting information

S1 Table Genetic Metrics. The results of several genetic metrics calculated for each brood year, including samples from the natural population (Nat), smolt release group (SRG), and adult release group (ARG). Values include the number of hybrids detected (and removed from further analyses), expected heterozygosity (*H*_*e*_), allelic richness (AR), the effective number of breeders per year (*N*_*b*_) with 95% confidence intervals (CI), and the effective population size (*N*_*e*_) as derived from *N*_*b*_. The first three populations represent those that underwent supplementation, while the latter three serve as control populations.
(DOCX)
