# Supplementary material for: Genetic monitoring of a long-term, large-scale experimental steelhead supplementation program
Source: PLoS One. 2025 Dec 22;20(12):e0339458. doi: 10.1371/journal.pone.0339458 (PMC12721512; doi:10.1371/journal.pone.0339458)
Supplement: S1 Table — The results of several genetic metrics calculated for each brood year, including samples from the natural population (Nat), smolt release group (SRG), and adult release group (ARG). Values include the number of hybrids detected (and removed from further analyses), expected heterozygosity (He), allelic richness (AR), the effective number of breeders per year (Nb) with 95% confidence intervals (CI), and the effective population size (Ne) as derived from Nb. The first three populations represent those that underwent supplementation, while the latter three serve as control populations. (DOCX) [file pone.0339458.s001.docx]

| Population /  Brood Year | Type | Hybrids | Final N | *H_e_* | AR | *N_b_* | Low 95% CI | High 95% CI | *N_e_* |
| --- | --- | --- | --- | --- | --- | --- | --- | --- | --- |
| Dewatto |  |  |  |  |  |  |  |  |  |
| 2004 | Nat | 1 | 21 | 0.750 | 4.42 | 18.4 | 10.1 | 43.7 | 75.4 |
| 2005 | Nat | 6 | 72 | 0.778 | 4.59 | 25.5 | 19.8 | 33.3 | 104.6 |
| 2006 | Nat | 2 | 178 | 0.797 | 4.59 | 55.5 | 46.7 | 66.4 | 227.6 |
| 2007 | Nat | 27 | 124 | 0.773 | 4.41 | 34.3 | 27.5 | 43.1 | 140.6 |
| 2007 | ARG | 2 | 27 | 0.774 | 4.50 | 32.6 | 19.4 | 72.2 | 133.7 |
| 2007 | SRG | 0 | 86 | 0.785 | 4.51 | 19.5 | 15.5 | 24.7 | 80.0 |
| 2008 | Nat | 3 | 18 | 0.767 | 4.56 | 19.8 | 5.8 | 1000 | 81.2 |
| 2008 | SRG | 0 | 90 | 0.742 | 4.21 | 21.2 | 17.7 | 25.5 | 86.9 |
| 2009 | Nat | 5 | 28 | 0.756 | 4.63 | 15.9 | 9.3 | 30.7 | 65.2 |
| 2009 | ARG | 1 | 256 | 0.729 | 4.15 | 6.0 | 4.0 | 7.4 | 24.6 |
| 2009 | SRG | 3 | 95 | 0.736 | 4.11 | 6.6 | 4.0 | 8.9 | 27.1 |
| 2010 | Nat | 4 | 44 | 0.784 | 4.50 | 46.4 | 31.4 | 77.1 | 190.2 |
| 2010 | SRG | 0 | 95 | 0.717 | 4.32 | 4.8 | 3.5 | 7.2 | 19.7 |
| 2011 | Nat | 8 | 66 | 0.789 | 4.55 | 78.8 | 53.8 | 131.1 | 323.1 |
| 2011 | ARG | 3 | 261 | 0.797 | 4.71 | 29.4 | 26.0 | 33.3 | 120.5 |
| 2011 | SRG | 1 | 95 | 0.776 | 4.65 | 23.0 | 18.6 | 28.5 | 94.3 |
| 2012 | Nat | 7 | 70 | 0.783 | 4.55 | 52.6 | 38.0 | 77.7 | 215.7 |
| 2012 | SRG | 0 | 94 | 0.758 | 4.26 | 17.5 | 14.8 | 20.6 | 71.8 |
| 2013 | Nat | 15 | 98 | 0.781 | 4.49 | 33.5 | 25.7 | 44.4 | 137.4 |
| 2013 | ARG | 0 | 261 | 0.751 | 4.17 | 31.8 | 27.9 | 36.3 | 130.4 |
| 2013 | SRG | 3 | 91 | 0.721 | 4.14 | 19.3 | 15.3 | 24.3 | 79.1 |
| 2014 | Nat | 8 | 126 | 0.779 | 4.50 | 43.9 | 34.7 | 56.3 | 180.0 |
| 2014 | SRG | 0 | 89 | 0.783 | 4.47 | 17.6 | 14.4 | 21.6 | 72.2 |
| 2015 | Nat | 4 | 98 | 0.780 | 4.42 | 116.5 | 84.4 | 175.3 | 477.7 |
| 2016 | Nat | 2 | 156 | 0.788 | 4.53 | 62.8 | 50.4 | 79.5 | 257.5 |
| 2017 | Nat | 17 | 299 | 0.768 | 4.39 | 70.3 | 59.1 | 83.9 | 288.2 |
| 2018 | Nat | 31 | 185 | 0.798 | 4.59 | 48.6 | 41.5 | 57.2 | 199.3 |
| 2019 | Nat | 35 | 129 | 0.775 | 4.46 | 25.7 | 19.8 | 33.4 | 105.4 |
| 2020 | Nat | 28 | 230 | 0.778 | 4.44 | 31.1 | 26.5 | 36.3 | 127.5 |
| 2021 | Nat | 15 | 52 | 0.754 | 4.33 | 21.7 | 15.5 | 31.2 | 89.0 |
| Duckabush |  |  |  |  |  |  |  |  |  |
| 2004 | Nat | 1 | 52 | 0.813 | 4.89 | 79.5 | 48.8 | 168.8 | 326.0 |
| 2005 | Nat | 2 | 90 | 0.809 | 4.84 | 54.1 | 40.6 | 75.1 | 221.8 |
| 2006 | Nat | 0 | 87 | 0.800 | 4.72 | 89.5 | 62.8 | 140.6 | 367.0 |
| 2007 | Nat | 4 | 76 | 0.823 | 4.94 | 47.5 | 34.4 | 69 | 194.8 |
| 2007 | ARG | 0 | 52 | 0.687 | 4.75 | 3.3 | 2.5 | 5.4 | 13.5 |
| 2007 | SRG | 0 | 92 | 0.765 | 4.55 | 3.0 | 2.5 | 3.6 | 12.3 |
| 2008 | Nat | 0 | 6 | 0.717 | 4.51 | 17.8 | 2.0 | ∞ | 73.0 |
| 2008 | ARG | 1 | 213 | 0.774 | 4.45 | 17.8 | 15.2 | 20.8 | 73.0 |
| 2008 | SRG | 5 | 90 | 0.766 | 4.64 | 11.4 | 9.3 | 13.8 | 46.7 |
| 2009 | Nat | 0 | 17 | 0.804 | 4.88 | 94.8 | 30.4 | ∞ | 388.7 |
| 2010 | Nat | 1 | 44 | 0.807 | 4.99 | 59.2 | 31.6 | 182.7 | 242.7 |
| 2010 | ARG | 0 | 206 | 0.738 | 4.74 | 6.3 | 4.4 | 7.7 | 25.8 |
| 2010 | SRG | 0 | 95 | 0.745 | 4.57 | 4.1 | 3.6 | 6.3 | 16.8 |
| 2011 | Nat | 1 | 109 | 0.811 | 4.85 | 51.6 | 38.4 | 71.7 | 211.6 |
| 2011 | SRG | 0 | 93 | 0.784 | 4.67 | 21.6 | 17.3 | 26.9 | 88.6 |
| 2012 | Nat | 0 | 27 | 0.807 | 4.90 | 114.3 | 52.6 | ∞ | 468.6 |
| 2012 | ARG | 3 | 233 | 0.795 | 4.80 | 11.9 | 10.0 | 13.9 | 48.8 |
| 2012 | SRG | 1 | 93 | 0.805 | 4.89 | 15.1 | 11.9 | 19.0 | 61.9 |
| 2013 | Nat | 1 | 34 | 0.809 | 4.87 | 96.2 | 45 | 1564.5 | 394.4 |
| 2013 | SRG | 0 | 95 | 0.803 | 4.72 | 27.3 | 22.9 | 32.6 | 111.9 |
| 2014 | Nat | 1 | 130 | 0.807 | 4.80 | 132.1 | 99.8 | 185.2 | 541.6 |
| 2014 | SRG | 0 | 91 | 0.756 | 4.45 | 10.7 | 8.7 | 13.1 | 43.9 |
| 2015 | Nat | 2 | 98 | 0.807 | 4.86 | 110.9 | 75.6 | 185.0 | 454.7 |
| 2016 | Nat | 3 | 149 | 0.813 | 4.82 | 103.3 | 83.0 | 132.2 | 423.5 |
| 2017 | Nat | 2 | 91 | 0.821 | 4.91 | 71.5 | 52.9 | 102.1 | 293.2 |
| 2018 | Nat | 1 | 205 | 0.790 | 4.77 | 128.1 | 100.8 | 168.1 | 525.2 |
| 2019 | Nat | 1 | 178 | 0.813 | 4.87 | 145.9 | 113.7 | 194.9 | 598.2 |
| 2020 | Nat | 0 | 84 | 0.797 | 4.83 | 112.8 | 71.9 | 217.7 | 462.5 |
| 2021 | Nat | 5 | 42 | 0.803 | 4.82 | 48.3 | 26.3 | 132.8 | 198.0 |
| South Fork Skokomish |  |  |  |  |  |  |  |  |  |
| 2004 | Nat | 0 | 20 | 0.749 | 4.35 | 118.4 | 33.8 | ∞ | 485.4 |
| 2005 | Nat | 0 | 155 | 0.766 | 4.38 | 293.1 | 211.0 | 455.7 | 1201.7 |
| 2006 | Nat | 0 | 188 | 0.771 | 4.37 | 247.2 | 177.5 | 380.3 | 1013.5 |
| 2007 | Nat | 0 | 132 | 0.773 | 4.42 | 184.8 | 110.0 | 424.5 | 757.7 |
| 2007 | ARG | 0 | 69 | 0.773 | 4.45 | 58.5 | 43.0 | 85.0 | 239.9 |
| 2007 | SRG | 0 | 94 | 0.776 | 4.52 | 46.6 | 35.3 | 63.5 | 191.1 |
| 2008 | Nat | 0 | 18 | 0.767 | 4.43 | -143.7 | 393.8 | ∞ | na |
| 2008 | SRG | 0 | 90 | 0.769 | 4.49 | 43.0 | 32.7 | 58.2 | 176.3 |
| 2009 | Nat | 0 | 24 | 0.774 | 4.53 | 160.1 | 47.5 | ∞ | 656.4 |
| 2009 | ARG | 0 | 265 | 0.756 | 4.27 | 43.8 | 38.1 | 50.4 | 179.6 |
| 2009 | SRG | 0 | 100 | 0.762 | 4.27 | 42.0 | 32.8 | 55.0 | 172.2 |
| 2010 | Nat | 0 | 45 | 0.772 | 4.49 | 96.4 | 49.5 | 432.9 | 395.2 |
| 2010 | SRG | 0 | 95 | 0.775 | 4.35 | 34.7 | 25.0 | 49.3 | 142.3 |
| 2011 | Nat | 2 | 90 | 0.776 | 4.46 | 132.4 | 92.7 | 214.1 | 542.8 |
| 2011 | ARG | 0 | 349 | 0.770 | 4.48 | 38.8 | 34.3 | 43.8 | 159.1 |
| 2011 | SRG | 0 | 93 | 0.758 | 4.28 | 32.3 | 24.4 | 43.6 | 132.4 |
| 2012 | Nat | 4 | 143 | 0.775 | 4.45 | 248.8 | 171.6 | 418.2 | 1020.1 |
| 2012 | SRG | 0 | 96 | 0.775 | 4.46 | 46.9 | 36.1 | 62.4 | 192.3 |
| 2013 | Nat | 1 | 266 | 0.770 | 4.41 | 251.4 | 194.3 | 341.2 | 1030.7 |
| 2013 | ARG | 0 | 189 | 0.768 | 4.42 | 35.1 | 29.0 | 42.5 | 143.9 |
| 2013 | SRG | 0 | 96 | 0.760 | 4.39 | 32.5 | 25.8 | 41.4 | 133.3 |
| 2014 | Nat | 2 | 251 | 0.775 | 4.45 | 324.6 | 244.1 | 462.9 | 1330.9 |
| 2014 | SRG | 0 | 95 | 0.746 | 4.36 | 31.2 | 23.1 | 42.8 | 127.9 |
| 2015 | Nat | 0 | 307 | 0.776 | 4.42 | 83.3 | 55.8 | 127.7 | 341.5 |
| 2016 | Nat | 0 | 389 | 0.770 | 4.35 | 162.3 | 67.4 | 622.7 | 665.4 |
| 2017 | Nat | 0 | 92 | 0.790 | 4.61 | 139.0 | 80.0 | 355.7 | 569.9 |
| 2018 | Nat | 2 | 178 | 0.776 | 4.44 | 83.7 | 56.2 | 133.9 | 343.2 |
| 2019 | Nat | 0 | 228 | 0.785 | 4.53 | 97.8 | 61.7 | 168.9 | 401.0 |
| 2020 | Nat | 0 | 135 | 0.784 | 4.52 | 148.8 | 89.8 | 317.2 | 610.1 |
| 2021 | Nat | 0 | 43 | 0.770 | 4.49 | 262.8 | 140.7 | 1359.1 | 1077.5 |
| Big Beef |  |  |  |  |  |  |  |  |  |
| 2004 | Nat | 1 | 21 | 0.738 | 4.48 | 7.5 | 3.1 | 15.0 | 30.8 |
| 2005 | Nat | 13 | 89 | 0.779 | 4.65 | 21.9 | 17.4 | 27.6 | 89.8 |
| 2006 | Nat | 33 | 125 | 0.792 | 4.58 | 37.5 | 29.1 | 48.8 | 153.8 |
| 2007 | Nat | 25 | 50 | 0.806 | 4.74 | 31.6 | 19.4 | 58.8 | 129.6 |
| 2008 | Nat | 6 | 46 | 0.767 | 4.79 | 9.7 | 5.6 | 15.1 | 39.8 |
| 2010 | Nat | 15 | 21 | 0.724 | 4.34 | 13.0 | 7.0 | 27.9 | 53.3 |
| 2011 | Nat | 49 | 34 | 0.768 | 4.78 | 8.9 | 3.8 | 16.4 | 36.5 |
| 2012 | Nat | 27 | 101 | 0.792 | 4.78 | 5.5 | 3.7 | 7.5 | 22.6 |
| 2013 | Nat | 15 | 42 | 0.690 | 4.86 | 7.6 | 4.1 | 11.0 | 31.2 |
| 2014 | Nat | 9 | 37 | 0.707 | 4.42 | 6.8 | 3.3 | 12.7 | 27.9 |
| 2015 | Nat | 10 | 64 | 0.772 | 4.80 | 15.1 | 10.6 | 21.4 | 61.9 |
| 2016 | Nat | 12 | 49 | 0.796 | 4.85 | 24.4 | 17.4 | 35.8 | 100.0 |
| 2017 | Nat | 19 | 41 | 0.776 | 4.61 | 15.9 | 11.0 | 23.5 | 65.2 |
| 2018 | Nat | 50 | 78 | 0.751 | 4.70 | 12.2 | 9.1 | 15.9 | 50.0 |
| 2019 | Nat | 55 | 28 | 0.807 | 4.86 | 47.4 | 26.0 | 144.6 | 194.3 |
| 2020 | Nat | 122 | 47 | 0.723 | 4.57 | 13.0 | 9.0 | 18.7 | 53.3 |
| 2021 | Nat | 44 | 12 | 0.673 | 4.47 | 2.5 | 1.1 | 17.8 | 10.3 |
| Little Quilcene |  |  |  |  |  |  |  |  |  |
| 2006 | Nat | 6 | 11 | 0.717 | 4.42 | 6.6 | 1.8 | ∞ | 27.1 |
| 2007 | Nat | 38 | 60 | 0.787 | 4.59 | 35.4 | 26.9 | 48.3 | 145.1 |
| 2008 | Nat | 6 | 15 | 0.740 | 4.42 | 55.4 | 19.4 | ∞ | 227.1 |
| 2009 | Nat | 14 | 21 | 0.797 | 4.97 | 17.3 | 7.3 | 78.5 | 70.9 |
| 2010 | Nat | 15 | 18 | 0.751 | 4.44 | 35.6 | 11.9 | ∞ | 146.0 |
| 2011 | Nat | 13 | 17 | 0.789 | 4.73 | 23.1 | 8.4 | ∞ | 94.7 |
| 2012 | Nat | 14 | 28 | 0.774 | 4.57 | 34.3 | 20.7 | 73.9 | 140.6 |
| 2013 | Nat | 20 | 45 | 0.788 | 4.59 | 37.4 | 26.8 | 56.1 | 153.3 |
| 2014 | Nat | 4 | 31 | 0.768 | 4.51 | 25.0 | 14.3 | 53.6 | 102.5 |
| 2015 | Nat | 25 | 36 | 0.773 | 4.52 | 11.7 | 7.0 | 19.0 | 48.0 |
| 2016 | Nat | 25 | 54 | 0.775 | 4.61 | 25.3 | 18.3 | 36.2 | 103.7 |
| 2017 | Nat | 23 | 20 | 0.777 | 4.82 | 13.5 | 7.7 | 27.3 | 55.4 |
| 2018 | Nat | 65 | 77 | 0.786 | 4.72 | 26.5 | 17.1 | 42.8 | 108.7 |
| 2019 | Nat | 50 | 54 | 0.746 | 4.44 | 11.8 | 7.5 | 17.7 | 48.4 |
| 2020 | Nat | 60 | 26 | 0.765 | 4.73 | 9.4 | 5.3 | 16.2 | 38.5 |
| 2021 | Nat | 81 | 21 | 0.740 | 4.34 | 24.1 | 10.9 | 142.4 | 98.8 |
| Tahuya |  |  |  |  |  |  |  |  |  |
| 2004 | Nat | 0 | 16 | 0.769 | 4.61 | 162.8 | 35.4 | ∞ | 667.5 |
| 2005 | Nat | 1 | 189 | 0.806 | 4.66 | 140.5 | 110.9 | 184.3 | 576.1 |
| 2006 | Nat | 1 | 127 | 0.808 | 4.69 | 165.2 | 112.0 | 281.6 | 677.3 |
| 2007 | Nat | 0 | 99 | 0.793 | 4.59 | 109.1 | 78.7 | 165.0 | 447.3 |
| 2008 | Nat | 1 | 18 | 0.767 | 4.50 | 41.8 | 14.5 | ∞ | 171.4 |
| 2009 | Nat | 3 | 28 | 0.788 | 4.57 | 44.8 | 22.7 | 189.5 | 183.7 |
| 2010 | Nat | 5 | 46 | 0.795 | 4.60 | 92.7 | 49.3 | 344.5 | 380.1 |
| 2011 | Nat | 1 | 81 | 0.802 | 4.63 | 73.4 | 53.2 | 109.1 | 300.9 |
| 2012 | Nat | 2 | 25 | 0.767 | 4.42 | 238.5 | 49.6 | ∞ | 977.9 |
| 2013 | Nat | 6 | 31 | 0.773 | 4.37 | 60.8 | 32.6 | 205.9 | 249.3 |
| 2014 | Nat | 1 | 111 | 0.791 | 4.59 | 75.5 | 57.0 | 104.3 | 309.6 |
| 2015 | Nat | 0 | 49 | 0.800 | 4.59 | 73.6 | 38.7 | 252.6 | 301.8 |
| 2016 | Nat | 4 | 90 | 0.787 | 4.55 | 84.5 | 62.5 | 121.9 | 346.5 |
| 2017 | Nat | 3 | 54 | 0.792 | 4.66 | 42.5 | 27.7 | 73.7 | 174.3 |
| 2018 | Nat | 4 | 135 | 0.795 | 4.71 | 46.9 | 38.2 | 58.1 | 192.3 |
| 2019 | Nat | 26 | 121 | 0.792 | 4.65 | 67.2 | 53.0 | 87.6 | 275.5 |
| 2020 | Nat | 9 | 116 | 0.799 | 4.64 | 58.3 | 45.6 | 76.4 | 239.0 |
| 2021 | Nat | 7 | 49 | 0.787 | 4.66 | 57.4 | 36.3 | 111.4 | 235.3 |
